# A Deep-Learning Aided Diagnostic System in Assessing Developmental Dysplasia of the Hip on Pediatric Pelvic Radiographs

**DOI:** 10.3389/fped.2021.785480

**Published:** 2022-03-08

**Authors:** Weize Xu, Liqi Shu, Ping Gong, Chencui Huang, Jingxu Xu, Jingjiao Zhao, Qiang Shu, Ming Zhu, Guoqiang Qi, Guoqiang Zhao, Gang Yu

**Affiliations:** ^1^The Children's Hospital, Zhejiang University School of Medicine, Hangzhou, China; ^2^National Clinical Research Center for Child Health, Hangzhou, China; ^3^Department of Orthopedics, The Children's Hospital, Zhejiang University School of Medicine, Hangzhou, China; ^4^Department of Neurology, The Warren Alpert Medical School of Brown University, Providence, RI, United States; ^5^Deepwise AI Lab, Beijing Deepwise & League of PHD Technology Co., Ltd., Beijing, China; ^6^Sino-Finland Joint AI Laboratory for Child Health of Zhejiang Province, Beijing, China

**Keywords:** developmental dysplasia of the hip, deep learning, three-stage pipeline, high-resolution network, aided diagnostic system

## Abstract

**Background:**

Developmental dysplasia of the hip (DDH) is a common orthopedic disease in children. In clinical surgery, it is essential to quickly and accurately locate the exact position of the lesion, and there are still some controversies relating to DDH status. We adopt artificial intelligence (AI) to solve the above problems.

**Methods:**

In this paper, automatic DDH measurements and classifications were achieved using a three-stage pipeline. In the first stage, we used Mask-RCNN to detect the local features of the image and segment the bony pelvis, including the ilium, pubis, ischium, and femoral heads. For the second stage, local image patches focused on semantically related areas for DDH landmarks were extracted by high-resolution network (HRNet). In the third stage, some radiographic results are obtained. In the above process, we used 1,265 patient x-ray samples as the training set and 133 samples from two other medical institutions as the verification set. The results of AI were compared with three orthopedic surgeons for reliability and time consumption.

**Results:**

AI-aided diagnostic system's Tönnis and International Hip Dysplasia Institute (IHDI) classification accuracies for both hips ranged from 0.86 to 0.95. The measurements of numerical indices showed that there was no statistically significant difference between surgeons and AI. Tönnis and IHDI indicators were similar across the AI system, intermediate surgeon, and junior surgeon. Among some objective interpretation indicators, such as acetabular index and CE angle, there were good stability and consistency among the four observers. Intraclass consistency of acetabular index and CE angle among surgeons was 0.79–0.98, while AI was 1.00. The measurement time required by AI was significantly less than that of the doctors.

**Conclusion:**

The AI-aided diagnosis system can quickly and automatically measure important parameters and improve the quality of clinical diagnosis and screening referral process with a convenient and efficient way.

## Highlights

- We proposed an AI system that can automatically report critical measurements for diagnosing developmental dysplasia of the hip with a performance similar to that of orthopedic surgeons, but require far less time, suggesting that it could have an important role in assisting the diagnosis of DDH.
- We designed clinical studies to validate the reliability and generalization performance of the AI system in calculating various parameters for diagnosing DDH.
- The algorithm framework presented in this study is of general significance for x-ray imaging-based orthopedic measurement.

## Introduction

Developmental dysplasia of the hip (DDH) is a common disorder that causes limb deformities. The incidence of hip joint instability after birth is 1%, and hip dislocation varies from 0.1 to 0.2% (1, 2). Anatomical defects of DDH is mainly the shallow acetabular depth, which makes the hip joint unstable (3). DDH is a recognized cause of secondary arthritis, which may lead to eventual total hip arthroplasty (THA) in order to relieve pain and improve function (4). However, the clinical symptoms of neonatal patient may be insignificant or only appear as a “clunks” sound when the hip is moving (5). Early recognition of DDH is associated with better outcomes (6).

X-ray is the most common method for diagnosing DDH at walking age, providing a vital role in DDH, such as acetabular index and center edge (CE) angle (7, 8). DDH treatment is related to the classification of Tönnis and IHDI, and the classification of IHDI and Tönnis plays an important role in determining the severity of DDH (9). However, the limitations of the current diagnostic to DDH mainly lower physician interpretation of diagnostic consistency and divergence. The study of Williams Daniel et al. demonstrated poor consistency of pediatric orthopedic surgeons in rating the 37 criteria for DDH (ICC, 0.39) (10). These problems affect the treatment and prognosis of children. Therefore, it is urgent to solve the problem of low consistency of DDH diagnostic process, and reduce the measurement errors and avoid the neglected cases for borderline acetabular dysplasia in massive screening.

Recently, several efforts were made to apply AI to DDH. Paserin et al. proposed a neural network that determines in real time whether the scanned 3D ultrasound image is suitable for diagnosis (11–13). These studies indicate that deep learning can accurately and robustly achieve automatic assessment of DDH on ultrasound images, and has great potential for clinical application. Bier et al. presented a method based on sequence prediction, which detected 23 key points to assist hip joint surgery decisions in complex scenarios (14). Chuanbin et al. adopted object detection to locate hip landmarks and calculate the acetabular index (7). Zhang (15) and Park (16) made use of convolutional neural network for detecting developmental development dysplasia of the hip. However, there are few artificial intelligence systems that can accurately measure hip x-ray and provide comprehensive DDH classification results.

To address the above problems, this research presents a deep-learning aided diagnostic system based on state-of-the-art deep learning technologies, which can automatically and reliably measure the acetabular index, CE angle, and provide Tönnis and IHDI classification results.

## Materials and Methods

**Figure 1:**
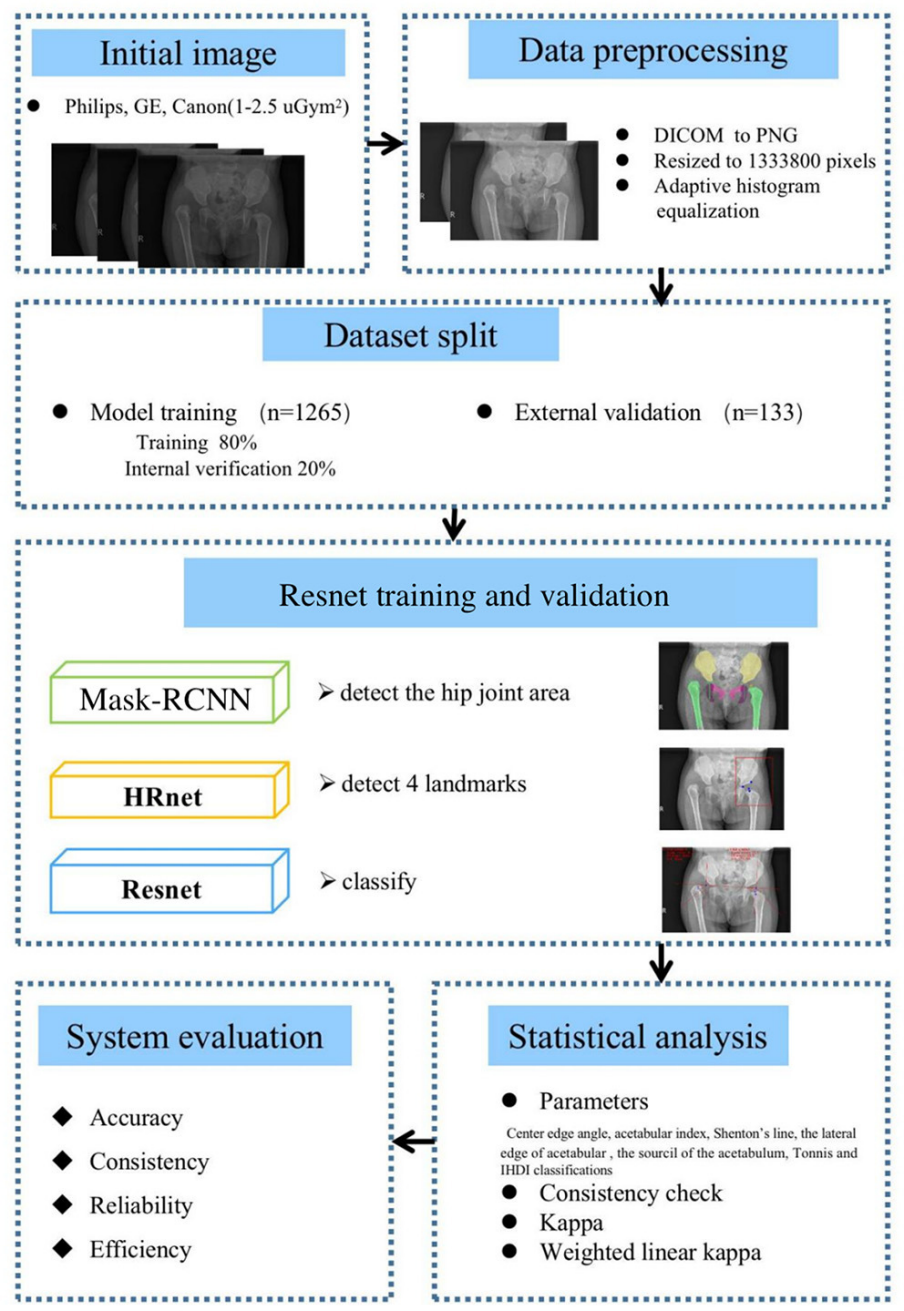
Method and analysis pipeline. Images were acquired at the hip joint, and all images were image preprocessed. The data set was divided into a model training set and an external verification set, Mask-RCNN, high-resolution network (HRNet), Resnet were used to gradually realize hip joint segmentation, key point positioning, and parameter measurement. The performance of the artificial intelligence (AI) system and doctors of different years of experience were compared and the accuracy, consistency, reliability, and efficiency of the AI system was evaluated.

An overview of the entire process is shown in Figure 1. This study was approved by the Medical Ethics Committee of the Children's Hospital (Approval Letter of IRB/EC, 2020-IRB-013) and waived the need for written informed consent from patients, as long as the data of the patient remained anonymous. All of the methods were carried out in accordance with the Declaration of Helsinki.

### Patients

The training set [1,265 cases from a hospital of children (Center 1)] and the verifying set [133 cases from other two hospitals of children (Center 2 & 3)] were annotated between June 2017 and February 2019 by the orthopedic senior surgeons with at least 10 years of experience. All patients had undergone an x-ray examination. Images for the patients were captured using Philips, GE, Canon. Exposure dose was 1–2.5 μGym^2^. X-ray images (1,398) of patients who underwent x-ray examinations were initially included in this study, based on the inclusion and exclusion criteria.

**Figure 2:**
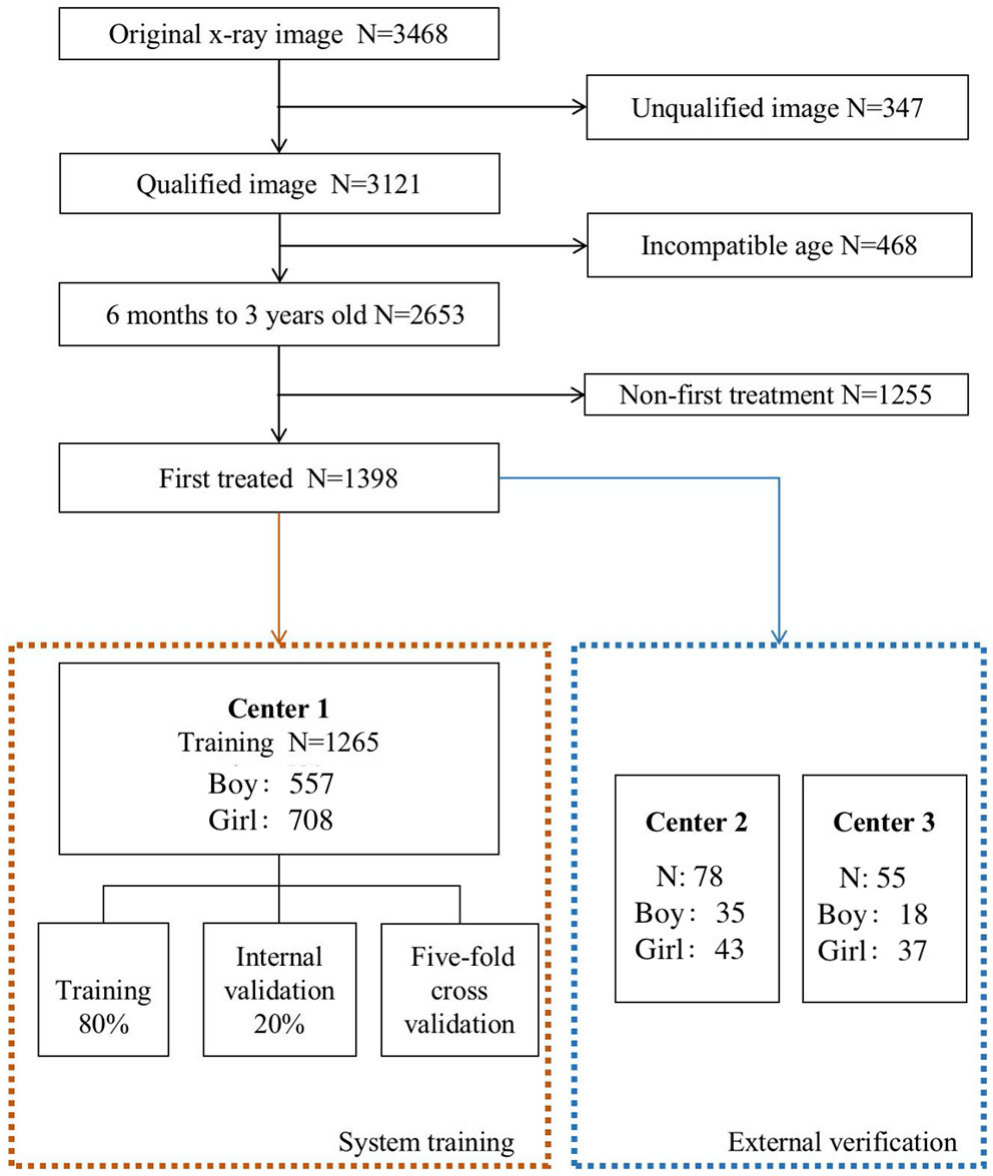
The process of including and excluding x-ray images. The 1,398 x-ray images [Center 1 (1,265), center 2 (78) and center 3 (55)] were selected from 3,468 original x-ray images as the research object.

As shown in Figure 2, there are 1,398 x-ray images finally incorporated into the AI system. Inclusion criteria are (1) 6 months and older, less than 3 years old; (2) the pelvic radiographs obtained must follow standard guidelines; (3) chief complaint of the visit is “checking for hip dysplasia”; and (4) x-ray image of DDH patient before first treatment. Exclusion criteria are (1) patients of hip dysplasia had been surgically treated; and (2) combined with other hip joint diseases, such as infection, femoral head Perthes disease, etc.

Standard pelvic radiograph requirements are as follows: (1) During the examination, the lower limbs are naturally straightened, the outside of the knees is flushed with the shoulders, the hips are slightly flexed, and the feet are taken in about 15°. (2) The size of the bilateral ilium and the obturator is basically symmetrical, the anterior and posterior edges of the acetabulum overlap, and the posterior margin of the acetabulum is not visible in x-ray image.

### Data Preprocessing

Anteroposterior pelvic radiographs were converted from DICOM format to PNG images using Python (version 3.6) and SimpleITK library (version 1.2.3). Images were resized to 1,333,800 pixels by keeping the original aspect ratio and padding zeroes on the shorter side. Resized images were further enhanced by applying window level and window width calculated by contrast-limited adaptive histogram equalization.

### Data Annotation

In our study, three radiologists annotated the contours of the pelvis bones, including the ilium, pubis, ischium, and femoral heads. Specifically, each sample was randomly assigned to a medium radiologist (with 5 years of experience) to obtain the preliminary labels. Then a senior radiologist (with more than 15 years of experience) checked and refined the preliminary labels to ensure the correctness of labeling.

### Model Implementation

**Figure 3:**
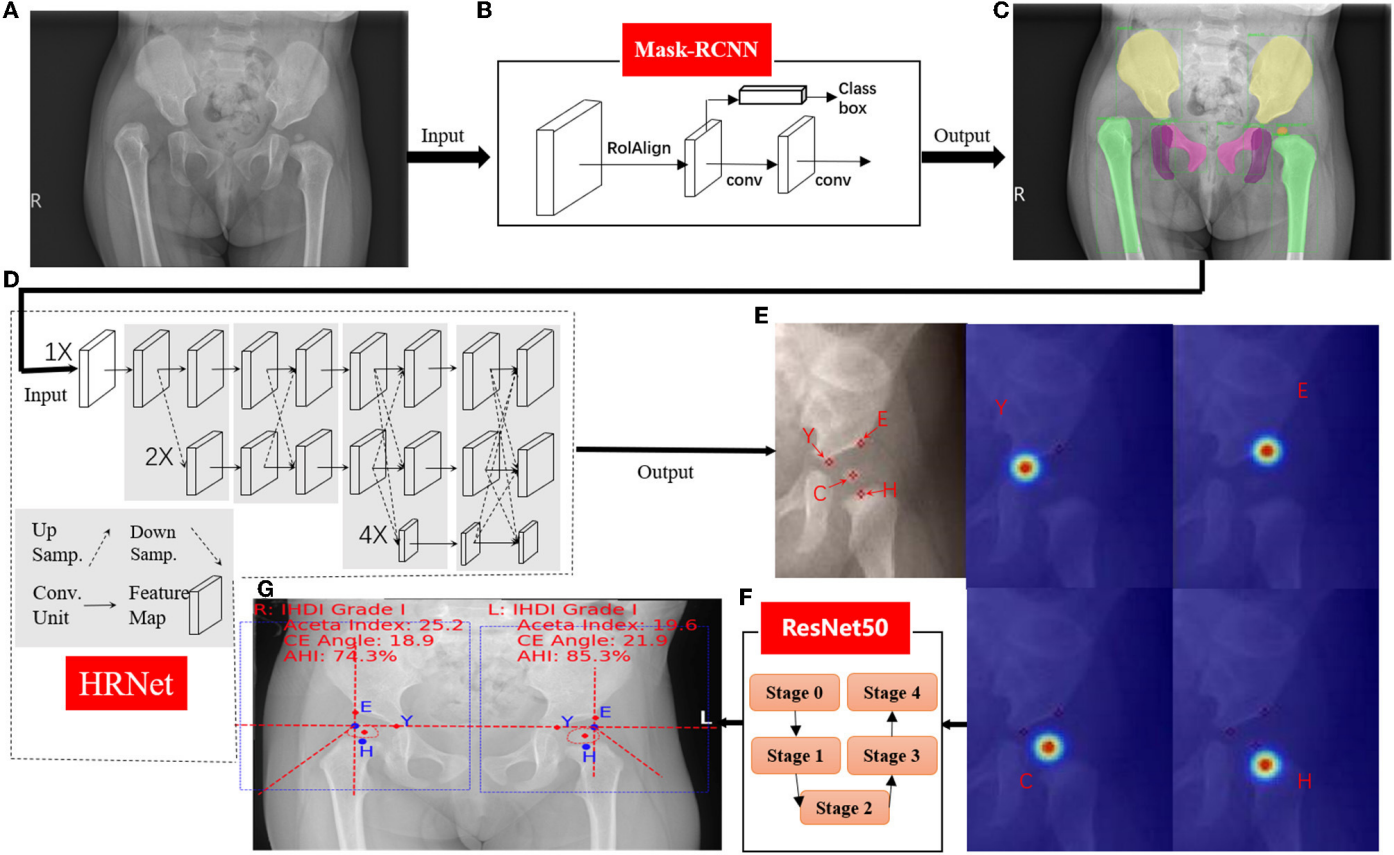
Hip joint segmentation and landmark detection heat map. **(A)** Hip joint x-ray initial image. **(B)** The flow-process diagram of Mask-RCNN. **(C)** The segmentation effect of the hip joint (different colors indicate different bones). **(D)** The flow-process diagram of HRNet. **(E)** Process heat map of four landmark extraction. **(F)** The flow-process diagram of ResNet50. **(G)** Angle and line measurements.

Automatic DDH measurements and classifications were achieved using a three-stage pipeline, as described below. The artificial intelligence model of the three-stage DDH measurement and classification is shown in Figure 3.

The first stage performed instance segmentation of pelvis bones, including the ilium, pubis, ischium, and the femoral heads. The femurs were also segmented for robust localization of (H) midpoint of the superior margin of the ossified femoral metaphysis, defined by the IHDI classification method (17). For instance, for segmentation, the Feature Pyramid Network (18) with ResNet50 (7) backbone was adopted, which was trained using stochastic gradient descent (SGD). Initial learning rate was 0.02, momentum was 0.9, and the weight decay was 0.0001. The segmentation results of this stage will be the basis of key point recognition in the second stage.

For the second stage, local image patches focused on semantically related areas for DDH landmarks were extracted. Within each patch, key point detection was performed to locate four pelvis landmarks: (E) the acetabulum superolateral margin, (Y) tri-radiate cartilage center, (C) femoral head center, and (H) midpoint of the superior margin of the ossified femoral metaphysis. We use HRNet (19) to identify the marked points, which can ensure that high resolution is maintained in the whole process of marker point recognition. Through parallel branches with multiple resolutions and information interaction between different branches, we can achieve the purpose of accurate location information and strong semantic information. HRNet occasionally generated false alarms of “point C” for infants, whose femoral heads were not visible on radiographs. To solve this issue, we utilized the robust femoral head segmentation results with high specificity produced by Mask-RCNN. Specifically, if the Mask-RCNN did not detect any ROI (region of interest) for femoral heads, the “point C” landmarks of the HRNet would be suppressed.

In the third stage, several radiographic measurements were derived. First, Hilgenreiner's line and Perkin's line were drawn. Then the acetabular index (20), the center edge angle (21), and the acetabular head index (22) were calculated. The Tönnis and IHDI method for DDH classification were automatically performed. This stage also output several qualitative judgments for each hip: whether the Shenton's line was intact or disrupted, whether the acetabulum superolateral margin was sharp or not, and whether the acetabular labrum was intact or not (23). Three multitask branches were appended to the ResNet architecture to learn those qualitative judgments. All models were trained with four TITAN Xp GPUs (Nvidia, Santa Clara, CA, USA).

### Statistical Analysis

Center edge angle, acetabular index, Shenton's line, the lateral edge of acetabular, the sourcil of the acetabulum, Tönnis, and IHDI classifications were useful for the diagnosis and treatment of DDH. In this research, we measured these seven parameters to compare the efficiency difference between surgeon and AI. Statistical differences were considered significant at *p* < 0.05.

Test–retest reliability of indicators of DDH diagnosis was assessed by three surgeons and AI a second time 2 weeks later. Three surgeons, including one senior surgeon, one intermediate surgeon, and one junior surgeon. Senior surgeons are deputy directors and above, intermediate surgeons are attending surgeons, and junior surgeons are resident surgeons. For reliability, consistency for the CE angle, and acetabular index were assessed using Cronbach's alpha (24). Alpha coefficients greater than or equal to 0.75 were considered satisfactory (25). Consistency check of subjective judgment indicators, such as Shenton's line, whether the lateral edge of acetabular is sharp or not, and whether the sourcil of the acetabulum is shallow or not, were statistically processed using kappa, and the Tönnis and IHDI classifications were the statistical processes using weighted linear kappa. A kappa statistic less than or equal to 0.40 was considered as indicating poor to slight agreement, 0.41–0.75 moderate agreement, and greater than 0.75 perfect agreement (26).

## Results

### Patient Demographics

The clinical records of patients were audited, including age and sex distribution. The age range of patients in this study was 6 months to 3 years. The average age of the training set was 1.184 ± 0.609 years. The average age of the test set was 1.193 ± 0.619 years. The number of boy and girl cases in the training set were 557 and 708, and in the test set were 53 and 80. There was no statistical difference in gender and age distribution between the two sets (*p* = 0.880, *p* = 0.355).

### Hip Joint Segmentation and Landmark Detection

As shown in Figure 3, Pyramid Network with ResNet50 was used to segment pelvis bones, including the ilium, pubis, ischium, and the femoral head for both hips. Then the four pelvis landmarks of the unilateral hip joint were detected: (E) the acetabulum superolateral margin, (Y) tri-radiate cartilage center, (C) femoral head center, (H) midpoint of the superior margin of the ossified femoral metaphysis. All parameters are measured based on the detection accuracy of landmarks. To evaluate landmark detection accuracy, the mean Euclidean distance error (MDE) between ground-truth landmark positions and the AI predicted landmark positions was used, and the results are summarized in Table 1. The high landmark detection accuracy has laid a good foundation for obtaining some classification and measurement indices of DDH.

**Table 1:** Measurement value of landmark detection accuracy.

**Landmark detection**	**Left hip**	**Right hip**
(E) The acetabulum superolateral margin	4.93	4.69
(Y) Tri-radiate cartilage center	5.37	5.21
(C) Femoral head center	4.62	4.20
(H) Midpoint of the superior margin of the ossified femoral metaphysis	4.15	4.06

### AI Model Effect Evaluation

**Figure 4:**
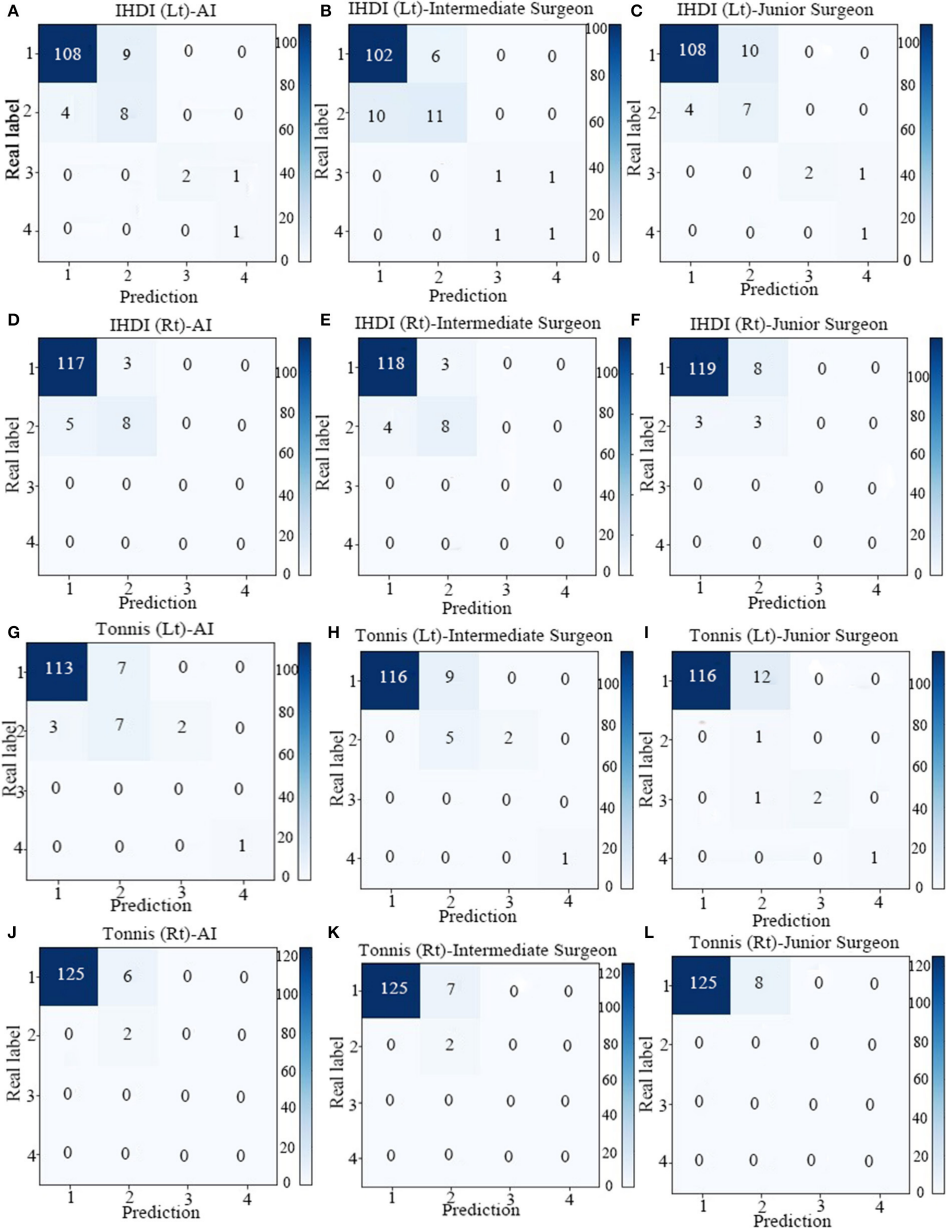
The confusion matrix of four indicators in AI system, Intermediate Surgeon and Junior Surgeon [IHDI (Lt), IHDI (Rt), Tonnis (Lt), Tonnis (Rt)]. **(A)** The confusion matrix of IHDI (Lt) in AI system. **(B)** The confusion matrix of IHDI (Lt) in Intermediate Surgeon. **(C)** The confusion matrix of IHDI (Lt) in Junior Surgeon. **(D)** The confusion matrix of IHDI (Rt) in AI system. **(E)** The confusion matrix of IHDI (Rt) Intermediate Surgeon. **(F)** The confusion matrix of IHDI (Rt) in Junior Surgeon. **(G)** The confusion matrix of Tonnis (Lt) in AI system. **(H)** The confusion matrix of Tonnis (Lt) in Intermediate Surgeon. **(I)** The confusion matrix of Tonnis (Lt) in Junior Surgeon. **(J)** The confusion matrix of Tonnis (Rt) in AI system. **(K)** The confusion matrix of Tonnis (Rt) in Intermediate Surgeon. **(L)** The confusion matrix of Tonnis (Rt) in Junior Surgeon.

A total of 113 pelvic x-rays as a validation set were analyzed. Taking consistent and undisputed data marked by senior surgeons in the test set as the standard, the accuracy, sensitivity, and specificity of the two-classification indices of AI were calculated, and the accuracy values of those indices ranged from 0.86 to 0.95, and the sensitivity values of those indices ranged from 0.84 to 0.96 (Table 2). The AI system was close to the intermediate surgeon, more sensitive than the junior surgeon, and has high specificity. The confusion matrix shows that the performances of Tönnis and IHDI indicators on the AI system, intermediate surgeon, and junior surgeon were similar (Figure 4).

**Table 2:** The accuracy, sensitivity, specificity, and missed diagnosis rate of six indicators in the artificial intelligence (AI) system, intermediate surgeon, and junior surgeon [Shenton's line (Lt), Shenton's line (Rt), lateral edge of acetabular (Lt), lateral edge of acetabular (Rt), sourcil of the acetabulum (Lt), sourcil of the acetabulum (Rt)].

**Indicators**	**Accuracy**			**Sensitivity**			**Specificity**		
	**AI**	**Intermediate surgeon**	**Junior surgeon**	**AI**	**Intermediate surgeon**	**Junior surgeon**	**Ai**	**Intermediate surgeon**	**Junior surgeon**
Shenton's Line (Lt)	0.917	0.902	0.835	0.92	0.909	0.864	0.911	0.889	0.778
Shenton's Line (Rt)	0.947	0.932	0.902	0.963	0.954	0.902	0.875	0.833	0.792
Lateral edge of acetabular (Lt)	0.887	0.872	0.774	0.868	0.857	0.736	0.929	0.904	0.857
Lateral edge of acetabular (Rt)	0.895	0.88	0.805	0.887	0.877	0.802	0.926	0.889	0.815
Sourcil of the acetabulum (Lt)	0.865	0.85	0.789	0.843	0.831	0.771	0.9	0.88	0.82
Sourcil of the acetabulum (Rt)	0.857	0.835	0.759	0.85	0.83	0.77	0.879	0.848	0.727

Mean CE angle (Lt) values in the surgeons and AI system were 18.00 ± 4.96 and 17.07 ± 6.71, respectively. There was no statistically significant difference between the surgeons and AI system (*p* = 0.743). The other three indicators like mean CE angle (Lt), acetabular index (Lt), and acetabular index (Rt) values had similar results: There was no statistically significant difference between surgeons and AI system (*p* = 0.483, *p* = 0.977, *p* = 0.131; summarized in Table 3).

**Table 3:** Measurement value and variance of four indicators in the AI system and surgeon [center edge (CE) angle (Lt), CE angle (Rt), acetabular index (Lt), acetabular index (Rt)].

**Indicators**	**Surgeons (Mean ± SD)**	**AI (Mean ± SD)**	***P* value**
CE Angle (Lt)	18.001 ± 4.955	17.074 ± 6.712	0.743
CE Angle (Rt)	21.902 ± 5.372	19.816 ± 6.883	0.483
Acetabular Index (Lt)	25.833 ± 4.095	25.883 ± 2.975	0.977
Acetabular Index (Rt)	22.733 ± 3.208	25.666 ± 4.533	0.131

### Test–Retest Reliability

**Figure 5:**
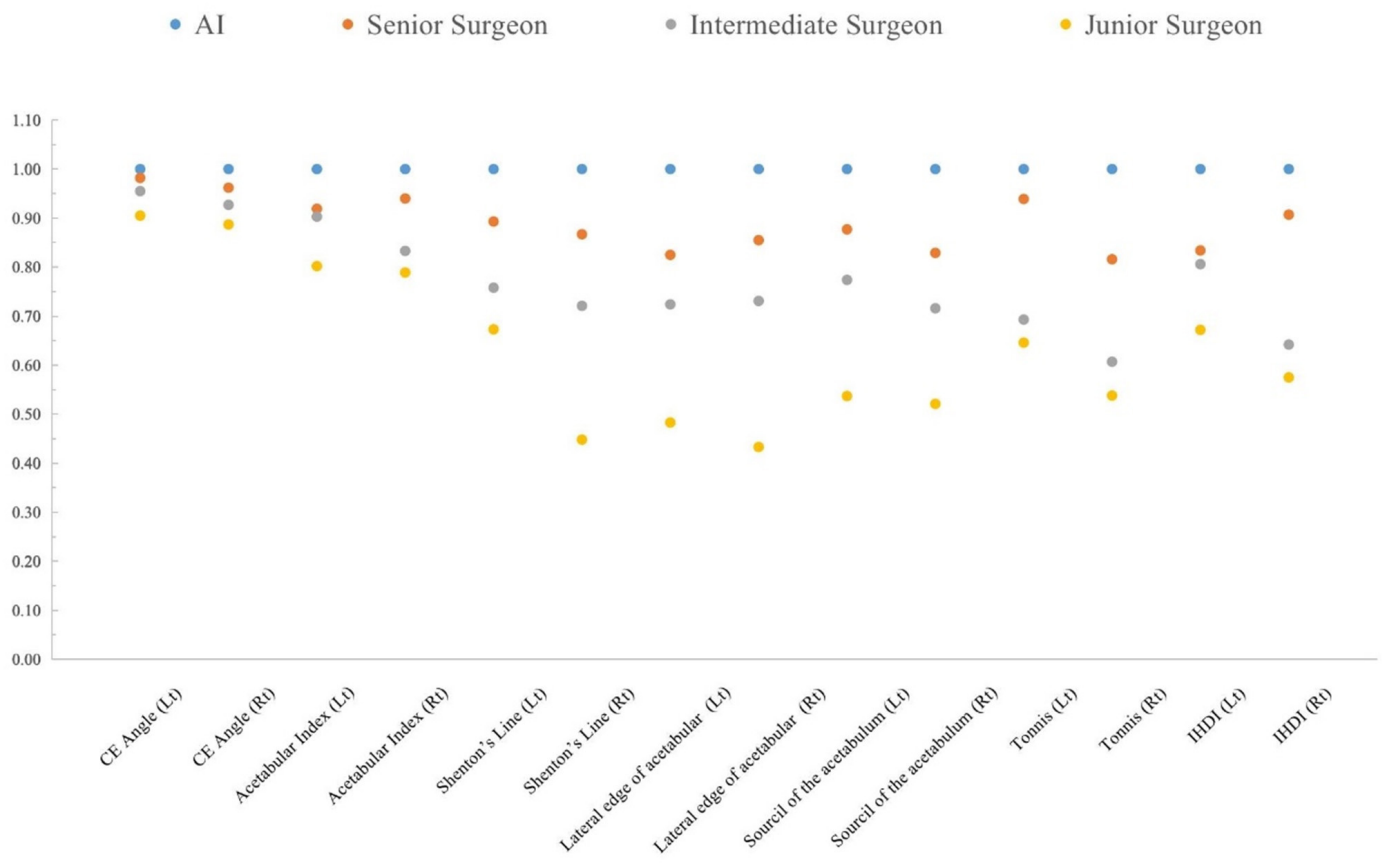
The intraclass consistency test of 14 indicators in the AI system, senior surgeon, intermediate surgeon, and junior surgeon.

In the AI group, all intraclass consistency results of the seven indicators showed extremely high stability and consistency. The intraclass consistency of the CE angle and acetabular index among the four groups all presented excellent agreement (>0.75). The intraclass consistency of Shenton's line, the lateral edge of acetabular, the sourcil of the acetabulum, Tönnis and IHDI classifications in the senior surgeon group performed well, which did not perform well in other surgeon groups. Figure 5 shows the results of intraclass consistency test.

### Time Consumption

Time consumption is the time consumed to obtain the seven DDH indicators for both surgeons and AI. The average time consumption was 1.21 ± 0.00 s per case for AI, while the average time for senior surgeon, intermediate surgeon, and junior surgeon were 150.36 ± 26.24, 200.71 ± 25.71, and 172.92 ± 20.58 s, respectively. The difference between AI and surgeons were significant (*p* < 0.001). Meanwhile, there were no significant differences in time consumption among surgeons (*p*-values were 0.773, 0.106, 0.249, respectively).

## Discussion

Early recognition of DDH is associated with better outcomes (27). Clearly defined, well formulated diagnostic criteria are vital to identify patients needing observation or treatment. In this study, we have incorporated objective parameters such as CE angle and acetabular index, the classification criteria of Tönnis and IHDI, exploring the diagnostic stability of surgeons to screen and highlight reliable indicators of diagnostic value. The Shenton's line, the outside boundary of the acetabulum whether sharp or not, the sourcil of the acetabulum whether shallow or not, are also being investigated in this study, as these parameters are inspirations for the diagnosis of DDH, while the effective usages are related to better clinical experience and work practice of the surgeon.

This paper presented an aided diagnostic algorithm framework based on deep learning technology, which can measure the acetabular index, CE angle, and calculate Tönnis grade and IHDI grade automatically, quickly, and accurately. During training, to balance the relatively few positive samples with common negative samples, weighted cross entropy losses were adopted. Experiments demonstrated that AI could achieve excellent stability in calculating various parameters for diagnosing DDH. Furthermore, orthopedic x-ray imaging diagnosis of various parts of the human body involves many angles and length measurements. For example, the humeral neck–shaft angle is measured for shoulder (28); the radial palmar tilt and radial back tilt are measured for the wrist (29). The accurate measurement of these angles has important implications for the diagnosis of fractures and dislocations. Therefore, the algorithm framework presented in this study is of general significance for orthopedic x-ray imaging measurement.

In this study, there were several indicators of intra-class consistency that presented low to moderate agreement, indicating the instability of these diagnostic parameters for DDH. The Shenton's line, the outside boundary of the acetabulum, and the sourcil of the acetabulum are subjective diagnostic indicators, and the judgment effect are related to clinical experience. It was also found that the inter-class consistency of Shenton's line was low (−0.01–0.33) (30). CE angle and acetabular index are the main reference indices for diagnosis of DDH, and from the results of this study, it has shown excellent consistency of surgeons whatever their working years are. In previous studies of Eitan Segev et al., the results showed that the acetabular index has a good consistency among different observers in the diagnosis of DDH, and affirmed the diagnostic value of CE angle and acetabular index (31, 32).

There are few limitations of this study. First, the interpretation of the hip joint is a systematic project; AI has obvious advantages in objective indicators, but it needs further learning and improvement in some indicators that involve subjectivity. Thus, the training set size should be increased in the future to improve the diagnostic ability of AI on subjective indicators. Second, the comprehensive improvement of model diagnostic performance needs to be closely integrated with clinical practice and application.

In summary, this study shows that the proposed AI-aided diagnostic system can automatically measure the results of the hip joint with a performance similar to that of orthopedic surgeons, with consistency and efficiency, suggesting that it could have an important role in assisting the diagnosis of DDH.

## Data Availability Statement

The datasets presented in this article are not readily available because the data set consists of juvenile X-ray data. In order to protect the privacy of minors, we will not disclose the dataset. Requests to access the datasets should be directed to Guoqiang Qi, qiguoqiang@zju.edu.cn.

## Ethics Statement

The studies involving human participants were reviewed and approved by Medical Ethics Committee, The Children's Hospital, Zhejiang University School of Medicine. Written informed consent from the participants' legal guardian/next of kin was not required to participate in this study in accordance with the national legislation and the institutional requirements.

## Author Contributions

WX and GY conceptualized and designed the study, performed the analysis, and drafted, reviewed, and edited the article. LS analyzed, reviewed, and critically revised the article for important intellectual content. PG analyzed and interpreted the data, trained the deep learning models, and reviewed and edited the article. CH, JX, and GQ analyzed and interpreted the data and reviewed and edited the article. JZ analyzed and interpreted the data. MZ and QS reviewed and edited the article. GZ reviewed and edited the article and agreed to be accountable for all aspects of the work if questions arise related to its accuracy or integrity. All authors contributed to the article and approved the submitted version.

## Funding

This study was supported by grants from the National Key Research and Development Program of China (Grant No. 2019YFE0126200), the National Natural Science Foundation of China (No. 62076218), and the Central Universities (Grant No. 2019XZZX003-16).

## Conflict of Interest

PG, CH, JX, and JZ are employed by Beijing Deepwise & League of PHD Technology Co., Ltd. The remaining authors declare that the research was conducted in the absence of any commercial or financial relationships that could be construed as a potential conflict of interest.

## Publisher's Note

All claims expressed in this article are solely those of the authors and do not necessarily represent those of their affiliated organizations, or those of the publisher, the editors and the reviewers. Any product that may be evaluated in this article, or claim that may be made by its manufacturer, is not guaranteed or endorsed by the publisher.
